# Using routine testing data to understand circulation patterns of influenza A, respiratory syncytial virus and other respiratory viruses in Victoria, Australia

**DOI:** 10.1017/S0950268819001055

**Published:** 2019-06-17

**Authors:** O. H. Price, S. G. Sullivan, C. Sutterby, J. Druce, K. S. Carville

**Affiliations:** 1 WHO Collaborating Centre for Reference and Research on Influenza, at the Peter Doherty Institute for Infection and Immunity, Victoria 3000, Australia; 2 School of Population and Global Health, University of Melbourne, Melbourne, Australia; 3 Victorian Infectious Diseases Reference Laboratory, at the Peter Doherty Institute for Infection and Immunity, Victoria 3000, Australia

**Keywords:** Co-infection, epidemiology, influenza, respiratory infections, respiratory syncytial virus

## Abstract

Several studies have reported evidence of interference between respiratory viruses: respiratory viruses rarely reach their epidemic peak concurrently and there appears to be a negative association between infection with one respiratory virus and co-infection with another. We used results spanning 16 years (2002–2017) of a routine diagnostic multiplex panel that tests for nine respiratory viruses to further investigate these interactions in Victoria, Australia. Time series analyses were used to plot the proportion positive for each virus. The seasonality of all viruses included was compared with respiratory syncytial virus (RSV) and influenza A virus using cross-correlations. Logistic regression was used to explore the likelihood of co-infection with one virus given infection with another. Seasonal peaks were observed each year for influenza A and RSV and less frequently for influenza B, coronavirus and parainfluenza virus. RSV circulated an average of 6 weeks before influenza A. Co-infection with another respiratory virus was less common with picornavirus, RSV or influenza A infection. Our findings provide further evidence of a temporal relationship in the circulation of respiratory viruses. A greater understanding of the interaction between respiratory viruses may enable better prediction of the timing and magnitude of respiratory virus epidemics.

## Introduction

Influenza, respiratory syncytial virus (RSV) and other respiratory viruses are the cause of substantial morbidity and mortality, with children under 5 years of age and the elderly disproportionately burdened [1]. Both influenza and RSV display distinct seasonality, however, the exact timing and magnitude of their annual epidemics remain difficult to predict [2]. A better understanding of the epidemiology of these pathogens is useful for the prevention and control of future epidemics and for optimising clinical management of patients [3]. Moreover, this knowledge may inform prediction models used to estimate the timing and magnitude of influenza epidemics [2].

Interference between respiratory viruses has been well documented. During peaks of influenza epidemics, the spread of other respiratory viruses, particularly RSV, appears to be limited [4–6]. Delays in outbreaks of influenza during the 2009 pandemic in Europe were linked to the annual rhinovirus epidemic associated with the beginning of the school year [7–9]. In turn, the influenza pandemic was observed to interfere with seasonal epidemics of RSV in France [10] and Israel [11], RSV and metapneumovirus in Germany [12], seasonal influenza in Hong Kong [13] and all respiratory viruses except rhinovirus in Beijing [14]. Studies investigating viral interference since the pandemic are sparser, though two studies reported that the timing and magnitude of respiratory virus epidemics were affected by the timing of the seasonal influenza A peak [15, 16]. Collectively, these observations suggest interference may prevent respiratory viruses reaching their epidemic peaks concurrently, but also underscore the complexity of these interactions.

The exact nature of interactions between different respiratory viruses remains unclear, although they are proposed to be driven by the innate immune system. Once a viral infection is established, interferon production is believed to confer temporary immunity to neighbouring cells against infection by other respiratory viruses [17]. In vitro, infection with RSV is blocked by competitive infection of influenza A if the host is not infected with the two viruses simultaneously [18]. Similarly, ferret models have shown that influenza A infection may prevent successive infection with RSV [19] and that coinfection with different influenza subtypes is dependent upon the order in which the viruses infect the host [20].

Despite this apparent interference, viral co-infections do occur, albeit with insufficient frequency to maintain an epidemic-level spread of the co-infecting viruses. A recent study reported infrequent co-detection of rhinovirus with other viruses [21], despite observations that rhinovirus continues to be shed for several weeks post-resolution of symptoms [22]. Negative associations have also been observed between the detection of influenza A, RSV, parainfluenza virus or coronavirus and co-detection of other respiratory viruses [8, 23, 24], providing further evidence for a refractory period after initial infection during which the host is less likely to be infected by subsequent exposure to another respiratory virus.

We used routine diagnostic testing data of specimens from both the community and hospitals at the Victorian Infectious Diseases Reference Laboratory (VIDRL) between 2002 and 2017 to describe relationships between respiratory viruses, with a focus on influenza A and RSV.

## Methods

### Clinical samples

From May 2002 to December 2017, 58 114 clinical specimens were collected from communities and hospitals and tested by polymerase chain reaction (PCR) for respiratory virus infection at VIDRL. There were no inclusion criteria regarding symptoms, but it is assumed that testing was deemed clinically relevant. Multiple specimen types were tested, but the majority were nose/throat swabs (64.2%) or nasopharyngeal aspirates (17.1%). The respiratory panel included nine viruses: adenovirus, influenza A, influenza B, parainfluenza virus, picornavirus (virus family includes rhinoviruses and enteroviruses), RSV, coronavirus (from 2010), human metapneumovirus (from 2012) and influenza C (from 2012). Data were de-identified, but the date of birth, postcode of residence and sex of the patient were provided.

### Data exclusion

Data exclusions are shown in Figure S1. Data from outbreaks, research and non-Victorian residents were excluded (*n* = 10 325) as they followed different sampling methods. Specimens collected from the same patient within 14 days were considered part of the same infection: where both specimens were positive for the same virus or both were negative, they were counted as one episode; where there were both positive and negative results, only the positive result was retained; and when two specimens were positive for different viruses, they were collapsed to represent one episode of co-detection. As a result, 8612 records were excluded. Data from 2009 (*n* = 4232) were excluded as the influenza pandemic led to changes in referral and testing practices. Data from 2016 to 2017 (*n* = 1293) were also excluded as the introduction of in-house testing at some referring hospitals led to a substantial decrease in samples tested by VIDRL.

### Statistical analysis

Demographic data of patients were compared using Pearson's *χ*^2^ test. Weekly proportions positive for each virus were calculated to allow comparability and assess differences in virus epidemics between seasons. We compared our data to influenza notification rates in Victoria obtained from the National Notifiable Diseases Surveillance System (NNDSS) [25] to assess the representativeness of inter-seasonal peaks we observed. To assess timing and magnitude of epidemics, the proportion of positive specimens and the peak week of the epidemic were considered: those in the lowest quartile were considered early or small and those in the highest quartile were considered late or large. Seasonality of viruses was assessed visually by time series analysis and for further investigation each virus was compared with influenza A and RSV using cross-correlations that estimated the association between peaks in epidemic curves at a lag or lead of up to 15 weeks.

Fisher's exact test was used to investigate any negative association between virus pairs among specimens with co-detections. Multivariate logistic regression, adjusted for age category (<5, 5–19, 20–64 and ⩾65 years), sex and season, was used to produce odds ratios (OR) and 95% confidence intervals for these associations and the chi-square test used to assess trend. Adjustment for multiple comparisons was not performed [23, 26]. The significance level for all tests was set at *P* < 0.05.

All data extraction, exclusion and analyses were performed in Stata (version 14.2, StataCorp, College Station, Texas).

## Results

### Respiratory virus detections

**Fig. 1.**
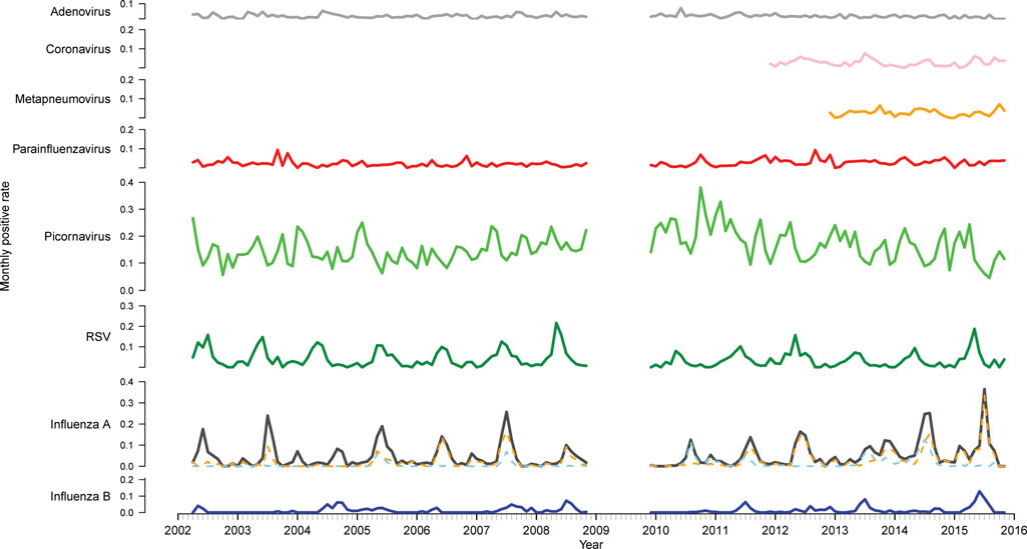
Time series of monthly virus detections. Data from 2009 were omitted as the influenza pandemic led to changes in referral practices. Influenza A is further divided into subtypes A(H3N2) (orange) and A(H1N1) (blue).

There were 33 652 PCR results from 2002 to 2015 (excluding 2009) included in this study. Of these, 11 154 (33.1%) were positive for at least one of the nine viruses tested for (Table 1). Picornavirus (rhinovirus) was detected most frequently (*n* = 5363, 33.1% of the positive specimens), followed by influenza A (*n* = 2259, 20.3%) and RSV (*n* = 1487, 13.3%). The proportion of tests positive for most viruses remained relatively stable (Fig. 1). However, there was a higher positivity rate of RSV pre-2009 (*P* < 0.001). The positivity rate of influenza A peaked and troughed; a year with a big epidemic was usually followed by a year with a smaller epidemic. Of the influenza A-positive samples, 57.9% were A(H3N2), 23.7% were A(H1N1) and the remaining not subtyped. In most seasons, one subtype predominated, although in 2005 and 2014, the subtypes were observed to circulate with similar magnitude and timing and in 2013 they circulated as two distinct peaks of comparable magnitude. The rate of picornavirus detection increased from 2006 to 2010 and then decreased from 2011 to 2015 returning to a level similar to that observed at the beginning of the study period.

**Table 1.** Demographic and temporal information for included specimens

	All (%)	Positive (%)	RSV (%)	Influenza A (%)	Influenza B (%)	Influenza C (%)	Picornavirus (%)	Adeno (%)	Parainfluenza (%)	Corona (%)	Metapneumovirus (%)
Total^a^	33 652	11 153 (33.1)	1487 (13.3)	2259 (20.3)	533 (4.8)	18 (0.3)^b^	5363 (48.1)	636 (5.7)	798 (7.2)	455 (6.8) ^b^	346 (8.2) ^b^
Age (years)											
Median	45.3	36.9	45.9	45.3	45.4	52.8	47.4	45.9	45.4	50.4	52.6
< 5	5714 (17.0)	2954 (26.5)	773 (52.0)	179 (7.9)	36 (6.8)	10 (55.6)	1651 (30.8)	363 (57.1)	249 (31.2)	67 (14.7)	51 (14.7)
5-19	1643 (4.9)	629 (5.6)	31 (2.1)	189 (8.4)	84 (15.8)	0 (0.0)	253 (4.7)	41 (6.4)	33 (4.2)	19 (4.2)	12 (3.5)
20-64	17 281 (51.3)	4799 (43.0)	388 (26.1)	1210 (53.6)	308 (57.8)	5 (27.8)	2165 (40.4)	175 (27.5)	349 (43.7)	247 (54.3)	155 (44.8)
65 years	7777 (23.1)	2171 (19.5)	244 (16.4)	672 (29.7)	103 (19.3)	3 (16.7)	773 (14.4)	31 (4.9)	161 (20.2)	112 (24.6)	127 (36.7)
Missing	1237 (3.7)	600 (5.4)	51 (3.4)	9 (0.4)	2 (0.4)	0 (0.0)	521 (9.7)	26 (4.1)	6 (0.8)	10 (2.2)	1 (0.3)
Sex											
Female	14 729 (43.8)	4821 (43.2)	665 (44.7)	1054 (46.7)	263 (49.3)	6 (33.3)	2111 (39.4)	273 (42.9)	355 (44.5)	219 (48.1)	174 (50.3)
Male	17 216 (51.2)	5492 (49.2)	751 (50.5)	1063 (47.1)	261 (49.0)	12 (66.7)	2673 (49.8)	335 (52.7)	433 (54.3)	218 (47.9)	156 (45.1)
Missing	1707 (5.1)	840 (7.5)	71 (4.8)	142 (6.3)	9 (1.7)	0 (0.0)	579 (10.8)	28 (4.4)	10 (1.3)	18 (4.0)	16 (4.6)
Residence											
Urban	25 502 (75.8)	8392 (75.3)	996 (67.0)	1790 (79.2)	415 (77.9)	16 (88.9)	4011 (74.8)	442 (69.5)	588 (73.7)	372 (81.8)	298 (86.1)
Rural	6248 (18.6)	2210 (19.8)	432 (29.1)	359 (15.9)	87 (16.3)	2 (11.1)	1048 (19.5)	164 (25.8)	178 (22.3)	76 (16.7)	42 (12.1)
Missing	1902 (5.7)	551 (4.9)	59 (4.0)	110 (4.9)	31 (5.8)	0 (0.0)	304 (5.7)	30 (4.7)	32 (4.0)	7 (1.5)	6 (1.7)
Season											
Winter	11 750 (34.9)	4668 (41.8)	987 (66.4)	1279 (56.6)	306 (57.4)	4 (22.2)	1614 (30.1)	255 (40.1)	213 (26.7)	224 (49.2)	152 (44.0)
Spring	9240 (27.5)	2931 (26.3)	187 (12.6)	684 (30.3)	167 (31.3)	12 (66.7)	1365 (25.5)	172 (27.0)	274 (34.3)	127 (27.9)	128 (37.0)
Summer	5598 (16.6)	1410 (12.6)	57 (3.8)	161 (7.1)	29 (5.4)	0 (0.0)	926 (17.3)	97 (15.3)	142 (17.8)	37 (8.1)	22 (6.4)
Autumn	7064 (21.0)	2144 (19.2)	256 (17.2)	135 (6.0)	31 (5.8)	2 (11.1)	1458 (27.2)	112 (17.6)	169 (21.2)	67 (14.7)	44 (12.7)

All percentages given are column percentages unless stated otherwise.

a Percentages for this row are row percentages. The percentage for positive tests is taken as a proportion of all tests; the percentage for each virus is taken as a proportion of positive tests.

b Denominator taken from samples from 2010 to 2015 for coronavirus and 2012–2015 for influenza C and human metapneumovirus

More specimens tested were collected from males (53.9%) (Table 1). As a proportion of total tests per sex, females were more likely to test positive for influenza A (*P* < 0.001) and metapneumovirus (*P* = 0.015), while males were more likely to test positive for picornavirus (*P* = 0.003) (Table S1). There was no significant difference in sex distribution for the other viruses. Patients residing in rural areas were significantly more likely to have a positive test than those in urban areas (*P* < 0.001). The same pattern was seen individually for RSV, parainfluenza virus and adenovirus. However, patients from urban areas were more likely to test positive for influenza A and metapneumovirus. Associations between remaining viruses and area of residence were not significant.

Respiratory virus tests were most frequently requested in winter (June–August; *n* = 11 750, 34.9%) (Table 1) and were most likely to be positive in winter (*P* < 0.001). Six of the nine viruses were most frequently detected in winter, but parainfluenza virus and metapneumovirus were most frequent in spring (September–November) and picornavirus was most frequent in autumn (March–May). Tests positive for picornavirus were distributed relatively evenly across the seasons, so although the modal week was in autumn, a peak was less distinct compared to other viruses.

The median age of positive tests was lower than that for all tests (36.9 (IQR: 2.4–61.5) and 45.3 (22.6–64.3) years, respectively). As a proportion of total tests per age group, children under 5 years had the highest burden of RSV, adenovirus, picornavirus and parainfluenza virus, while those aged 5–19 years had the highest burden of influenza types A and B and those aged 65 years and older had the greatest burden of metapneumovirus (Table S1). Notably, the median age of patients from whom specimens were collected increased fairly steadily from 27.6 years in 2002 to 61 years in 2015 (Table S2).

### Seasonality of viruses

**Fig. 2.**
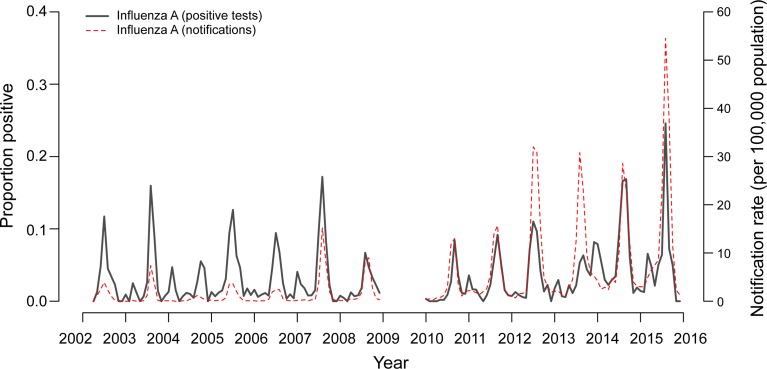
Two-week moving averages of weekly positive rates for influenza A compared to Victorian notification rates. Notification rates during seasons with high incidence of influenza (June–September 2014 and 2015) were scaled down 2:1 to allow better visualisation of inter-epidemic peaks. Data from 2009 were omitted as the influenza pandemic resulted in changed referral patterns.

Time series analysis demonstrated annual seasonal peaks for influenza A and RSV (Fig. 1). Peaks occurred most frequently in winter, with occasional peaks in late autumn (RSV) and early spring (influenza A). Although influenza A virus circulation during summer in Victoria is expected to be minimal, we observed increased influenza positivity rates in many summers during the study period, one of which was larger than its preceding winter peak (2013–2014). These inter-epidemic peaks were reflected in Victorian notification rates (obtained from NNDSS) in summers from 2010 to 2011 onwards, visible as influenza activity not reaching zero as it had in previous summers (Fig. 2). Seasonal peaks were also observed among the other viruses, except picornavirus, although they did not occur every year. Picornavirus remained endemic throughout the year for the duration of the study period.

Cross-correlations were performed to ascertain whether the timing and magnitude of other viruses may differ relative to influenza A activity. Results revealed a moderate to strong correlation between epidemic curves of influenza and RSV in 9 of 13 years. On average, where correlated, a seasonal epidemic of RSV occurred 6 weeks earlier than that of influenza A (Table 2), although there were 3 years where the epidemics occurred at similar times (2002, 2005 and 2006). As a sensitivity analysis, we performed further cross-correlations to assess whether influenza subtype affected these interactions (Table S3). The lag calculated for influenza A overall was consistently similar to that of the predominant influenza A subtype in a given season. In some years the lag calculated suggested influenza A(H1N1) circulated prior to RSV, however in these years the number of samples positive for influenza A(H1N1) was <10.

**Table 2.** Cross-correlation, timing and magnitude of influenza A and RSV epidemic curves, 2002–2015.

		Influenza A			RSV		
Year	Lag (coefficient)^a^	Peak week	Timing^b^	Magnitude^b^	Peak week	Timing^b^	Magnitude^b^
2002	**1** (**0.618)**	27	Early		32	Late	Large
2003	**−7 (0.668)**	32		Large	30	Late	
2004	−15 (0.377)	40	Late	Small	25		
2005	**−1 (0.757)**	27	Early		27		
2006	**−1 (0.677)**	30			30	Late	
2007	**−3 (0.726)**	32		Large	28		Large
2008	**−10 (0.701)**	35		Small	25		Large
2010	**−11 (0.633)**	36	Early	Small	25		Small
2011	**−10 (0.632)**	36	Late		27		Small
2012	−3 (0.467)	26	Early		23	Early	
2013	10 (−0.42)	35			29		Small
2014	**−12 (0.672)**	34		Large	22	Early	Small
2015	−7 (0.528)	31		Large	25	Early	Large

Correlations considered moderate (>0.6) or strong (>0.7) are bolded.

a Lag in weeks for RSV compared to influenza A, i.e. a negative number indicates RSV preceded influenza A.

b For timing and magnitude of epidemic curves, proportion of specimens positive and the peak week of the epidemic were considered: those in the highest quartile were considered late or large; those in the lowest quartile were considered early or small.

No consistent pattern emerged when considering timing and magnitude of influenza A and RSV seasons (Table 2): an early epidemic of one virus sometimes resulted in a later than usual epidemic of the other, but this was not always the case. Likewise, a season with a high magnitude of infections with one virus did not necessarily result in a season with a low magnitude of the other. Generally, influenza B epidemics occurred at a similar time to influenza A and in years that influenza A circulated early (2002, 2005, 2011, 2012), influenza B activity was low (data not shown).

### Co-detections

Co-detections of respiratory viruses occurred in 6.4% (*n* = 823) of positive samples. Exploratory data analysis using univariate logistic regression suggested co-detections were more likely to occur in children under 5 years, males and during winter. Odds of co-detection decreased as age increased. Using the <5 year age group as the reference category, the ORs (adjusted for sex and season) and corresponding 95% confidence intervals for co-infection were 0.37 (0.27–0.52) for those aged 5–19 years, 0.27 (0.23–0.33) for those aged 20–64 years and 0.19 (0.15–0.25) for those aged ⩾65 years (*P*-value for trend:  < 0.001).

**Fig. 3.**
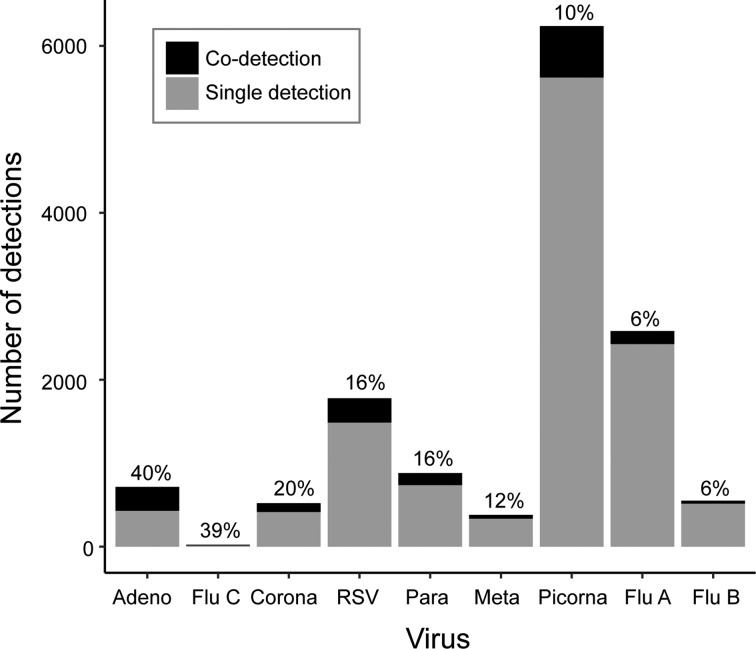
Viral co-detections.

Co-detections occurred most frequently with adenovirus (40%), influenza C (39%) and coronavirus (20%), though the number of influenza C infections was small (*n* = 18) (Fig. 3). Co-detections were rarest with picornavirus (10%) and influenza A and B (6%) infections. Analysis of co-infections using Fisher's exact test found a pattern of a negative association between detection of influenza A, RSV or picornavirus and co-detection of another virus (Table 3). These three viruses were involved in the highest number of significant negative associations (*n* = 5, Table 3). No positive associations between viruses were considered statistically significant. Multivariate logistic regression (adjusted for age group, sex and season) was used to further investigate the probability of co-detection given infection with influenza A, RSV and picornavirus (Table 4). Significant negative associations were observed for co-detection with all viruses where influenza A was detected and all but one virus for RSV and picornavirus detections (influenza B and human metapneumovirus, respectively).

**Table 3.** The probability of infection with each virus given infection with the other

	Adenovirus	Coronavirus	Influenza A	Influenza B	Influenza C	Metapneumovirus	Parainfluenza virus	Picornavirus	RSV
Adenovirus		**<0**.**001**	**<0**.**001**	**0**.**004**	0.868	**0**.**013**	**<0**.**001**	0.075	**<0**.**001**
		**0.35 (0.20–0.61)**	**0.11 (0.06–0.21)**	**0.17 (0.05–0.58)**	0.87 (0.18–4.30)	**0.26 (0.09–0.75)**	**0.24 (0.15–0.40)**	0.74 (0.54–1.03)	**0.26 (0.18–0.36)**
Coronavirus			**0.007**	0.255	0.763	**0.003**	0.144	**<0.001**	0.05
			**0.37 (0.18–0.76)**	1.66 (0.69–4.01)	0.78 (0.16–3.85)	**0.17 (0.05–0.55)**	0.64 (0.36–1.16)	**0.21 (0.13–0.33)**	0.58 (0.33–1.00)
Influenza A				**0.04**	^a^	0.094	**0.012**	**<0.001**	**<0.001**
				**0.12 (0.02–0.91)**	^a^	0.46 (0.19–1.14)	**0.49 (0.28–0.85)**	**0.37 (0.25–0.53)**	**0.37 (0.24–0.57)**
Influenza B					^a^	^a^	0.053	**<0.001**	0.146
					^a^	^a^	0.14 (0.02–1.03)	**0.25 (0.12–0.50)**	0.55 (0.25–1.23)
Influenza C						0.733	0.539	0.898	0.387
						0.69(0.08–5.68)	0.52 (0.06–4.23)	1.10 (0.27–4.48)	0.40 (0.05–3.23)
Metapneumovirus							**0.03**	0.519	**0.017**
							**0.26 (0.08–0.88)**	1.25 (0.63–2.47)	**0.27 (0.09–0.80)**
Parainfluenza virus								**<0.001**	**<0.001**
								**0.34 (0.23–0.49)**	**0.23 (0.14–0.39)**
Picornavirus									**<0.001**
									**0.34 (0.24–0.47)**
RSV									

Associations considered significant are bolded.

The top cell represents the *P*-value for each measure of association and the bottom cell the OR (and corresponding 95% CI) for infection.

a No co-detections with these two viruses occurred

**Table 4.** Multivariate logistic regression adjusted for sex, age group and season estimating the OR for co-infection with one virus given infection with another

Exposure	Outcome	OR (95% CI)	*P* -value
Influenza A	Influenza B	**0.04 (0.01–0.32)**	**0.002**
	RSV	**0.39 (0.24–0.63)**	**<0.001**
	Adenovirus	**0.18 (0.09–0.34)**	**<0.001**
	Parainfluenza virus	**0.36 (0.20–0.66)**	**0.001**
	Coronavirus	**0.25 (0.11–0.54)**	**<0.001**
	Human metapneumovirus	**0.34 (0.12–0.95)**	**0.040**
	Picornavirus	**0.46 (0.30–0.69)**	**<0.001**
RSV	Influenza A	**0.39 (0.25–0.63)**	**<0.001**
	Influenza B	0.62 (0.26–1.48)	0.285
	Adenovirus	**0.15 (0.10–0.22)**	**<0.001**
	Parainfluenza virus	**0.26 (0.15–0.43)**	**<0.001**
	Coronavirus	**0.51 (0.28–0.94)**	**0.030**
	Human metapneumovirus	**0.29 (0.10–0.87)**	**0.027**
	Picornavirus	**0.34 (0.24–0.49)**	**<0.001**
Picornavirus	Influenza A	**0.46 (0.30–0.69)**	**<0.001**
	Influenza B	**0.36 (0.17–0.77)**	**0.008**
	RSV	**0.34 (0.24–0.49)**	**<0.001**
	Adenovirus	**0.45 (0.31–0.66)**	**<0.001**
	Parainfluenza virus	**0.25 (0.16–0.38)**	**<0.001**
	Coronavirus	**0.24 (0.14–0.40)**	**<0.001**
	Human metapneumovirus	1.26 (0.60–2.64)	0.536

Associations considered significant are bolded.

## Discussion

We used multi-year routine PCR testing data to establish patterns of respiratory virus circulation in Victoria, Australia. Picornavirus (rhinovirus) was most frequently detected. Children aged <5 years and those living in rural areas experienced a high burden of infection. Time series analyses indicated the annual occurrence of epidemics for influenza A and RSV and less recurrent epidemics for influenza B, coronavirus and parainfluenza virus. Picornavirus was observed to be endemic throughout the period of analysis. RSV epidemics generally began in autumn and peaked early winter, while influenza A began mid-winter and peaked late winter. The higher incidence of RSV observed pre-2009 may be a result of the higher proportion of children under 5 years in our sample pre-2009, as RSV is considered the most important respiratory illness-causing pathogen in infants [27]. We observed summer peaks of influenza A in some years which was somewhat unexpected in a temperate climate but was only reflected in state-wide notification data after 2009. It is possible that inter-epidemic peaks we observed are a result of denominator data, while the increase in notifications resulted from a rise in testing after the 2009 pandemic [28]. In years that epidemics occurred, influenza B, coronavirus and parainfluenza virus peaked in winter and metapneumovirus in spring.

Like previous studies [15, 16], time series analyses and cross-correlations established distinct circulation patterns of RSV and influenza A. The two viruses rarely reached their epidemic peak concurrently, with RSV peaking an average of 6 weeks before influenza A. Influenza A subtype did not affect cross-correlations: in seasons where significant correlation was observed, the lag calculated for influenza A overall was similar to that of the predominant subtype. In some seasons, influenza A(H1N1) appeared to circulate prior to RSV. However, in these seasons the number of A(H1N1) positive samples was <10, so the results should be interpreted with caution. An investigation into seasonal relationships between epidemic curves of other viruses was limited by the small proportion of positive tests. The endemic nature of picornavirus appeared to be unaffected by the circulation of other respiratory viruses, which supports previous observations of rhinovirus activity (most common species of picornavirus) [15, 16]. This may be a result of increased stability of the non-enveloped picornavirus during warmer months compared to other viruses, like influenza, which are restricted by temperature and humidity [29].

We also investigated the distribution and incidence of respiratory virus co-detection. Improved availability and sensitivity of diagnostic tests has resulted in more regular detection of co-infections [30], though the impact of viral co-infection on clinical severity remains unclear [31, 32]. A prospective household transmission study during the 2009 influenza pandemic reported that the infection wave caused by influenza A(H1N1)pdm09 was interrupted by a wave of non-influenza respiratory virus infections [33]. It found individuals infected with influenza A(H1N1)pdm09 were less likely to be infected by non-influenza respiratory viruses than non-infected individuals (RR: 0.32). Further, there was a significant decrease in the duration of viral shedding in co-infections (of any respiratory viruses) compared to single infections. These observations suggest that such interactions may modulate influenza attack rate during outbreaks, thus shaping the epidemic and highlighting the importance of better understanding co-infections in the context of viral interference.

We found co-detections of respiratory viruses in 6.4% of positive specimens, which falls in the 5.0–62.0% range of previous studies [31]. Like other studies, we found co-infection was less likely with increasing age [34, 35], which may be a consequence of pre-existing immunity or decreased viral shedding with increasing age [30]. We found adenovirus and coronavirus most likely to be part of a co-infection and influenza A and B least likely, corroborating results of a previous study [36]. While our data did not include patients' symptoms, immunological [37] and clinical [24, 38] data suggest that the effect of co-infection on clinical severity depends on the specific pathogens co-infecting the patient.

Infection by rhinovirus may result in temporary immunity of the host to infection by other respiratory viruses due to the production of cytokines [17], thus resulting in a negative association between rhinovirus infection and co-infection with another virus [23]. Moreover, it is believed to be the driver of epidemiological interaction between respiratory viruses at the population level, which is visible when two viruses may not reach their epidemic peak during the same period. While rhinovirus has been the focus of other studies investigating co-detection, we found that influenza A, RSV and picornavirus all had significant negative associations with co-detection of other viruses. Results of further investigation by logistic regression adjusted for covariates that are predictors of codetection (sex, age and season) were compatible with influenza A, RSV and picornavirus conferring temporary immunity against infection by another respiratory virus. However, we cannot make causal inferences from the design used and therefore, cannot eliminate the role of other environmental factors.

Our study has some limitations. All inferences we made and indeed the majority of inferences made in other studies investigating respiratory virus interference are based on ecological data. With such data, we cannot determine whether events observed are the result of a biological mechanism, nor can we infer the direction of the postulated interaction, i.e. which virus impacts which. To make any form of causal inference, a prospective study that serially samples participants over multiple respiratory virus epidemics would be required. Furthermore, we did not adjust for other potential drivers of viral interference, such as environmental (e.g. temperature, humidity), social and behavioural factors. Also, we cannot rule out the possibility that our observations were the result of surveillance artefacts, that is, changes in testing patterns that are not a result of genuine fluctuations in viral circulation. While we excluded specimens isolated from outbreaks or for surveillance and samples from 2009 and 2016–2017 when testing patterns were obviously altered, we cannot be certain we controlled completely for this unknown. Type/subtype data for other viruses may have also improved the resolution of our findings, as other studies have noted variances in the timing of epidemics caused by different types of parainfluenza virus [4, 6] and rhinovirus [21] and there is scant information available for RSV. Additionally, our study sample was drawn from patients ill enough to seek healthcare. As some viruses (such as picornavirus) are more likely to result in asymptomatic infection than others, the distribution of viruses in our sample may differ from that in the population. Finally, the referral base of paediatric samples for VIDRL is limited as most Victorian paediatric samples are forwarded to a children's hospital. Given there is a high burden of respiratory virus infection in children, this may have limited our analyses.

A strength of our data is that it spanned 16 years. Accurately monitoring seasonal variation in respiratory virus epidemics has the potential to improve our understanding of interaction and interference between different respiratory viruses, although this remains challenging as surveillance systems for non-influenza respiratory viruses are limited in both scope and funding. Our study confirms the existence of temporal relationships in the circulation of some respiratory viruses in Victoria and provides further evidence to support the postulated effects of viral interference on magnitude and timing of respiratory virus epidemics.

## Supplementary material

For supplementary material accompanying this paper visit https://doi.org/10.1017/S0950268819001055. click here to view supplementary material
