# Supplementary material for: Using routine testing data to understand circulation patterns of influenza A, respiratory syncytial virus and other respiratory viruses in Victoria, Australia
Source: Epidemiol Infect. 2019 Jun 17;147:e221. doi: 10.1017/S0950268819001055 (PMC6625191; doi:10.1017/S0950268819001055)
Supplement: Supplementary file 1 [file S0950268819001055sup001.doc]

Epidemiology and Infection

Using routine testing data to understand circulation patterns of influenza A, respiratory syncytial virus and other respiratory viruses in Victoria, Australia

O.H. Price, S.G. Sullivan, C. Sutterby, J. Druce, K.S. Carville

Supplementary material

**Supplementary Figure S1** Included data


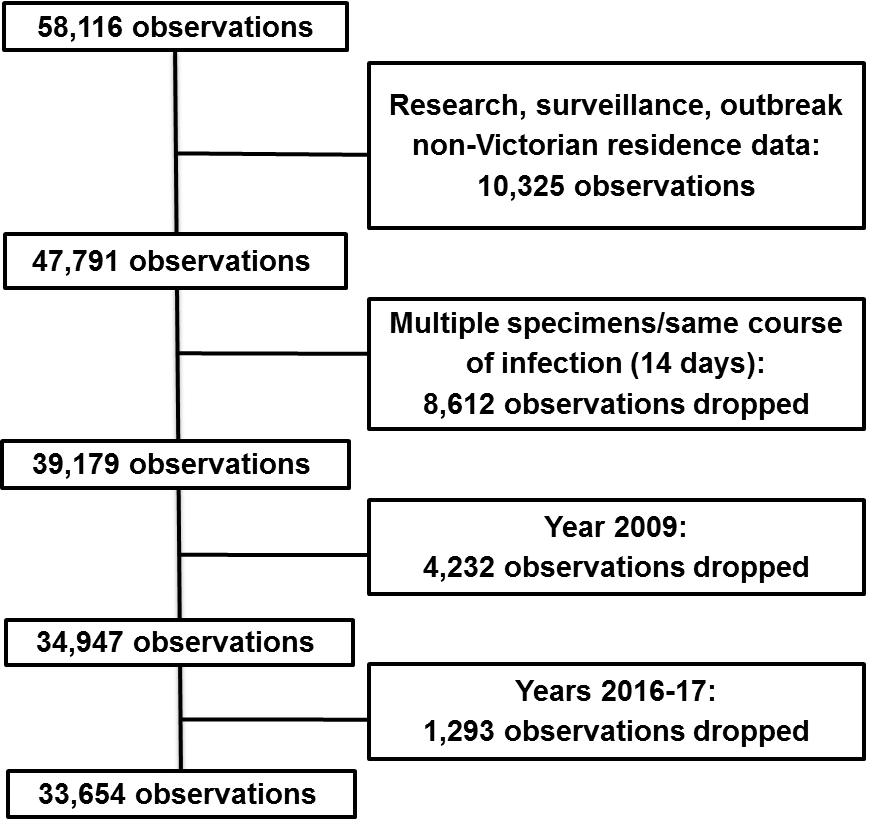


**Supplementary Table S1** Change in age distribution of specimens tested 2002-2015

| Age (years) | 2002 (%) | 2003 (%) | 2004 (%) | 2005 (%) | 2006 (%) | 2007 (%) | 2008 (%) | 2010 (%) | 2011 (%) | 2012 (%) | 2013 (%) | 2014 (%) | 2015 (%) |
| --- | --- | --- | --- | --- | --- | --- | --- | --- | --- | --- | --- | --- | --- |
| Median | 27.6 | 35.7 | 38.0 | 42.7 | 41.1 | 38.3 | 41.8 | 43.7 | 47.6 | 51.2 | 49.9 | 53.1 | 61.3 |
| <5 | 344 (38.6) | 397 (27.7) | 381 (28.1) | 399 (17.8) | 499 (22.8) | 754 (21.7) | 787 (23.8) | 619 (20.6) | 453 (14.3) | 336 (11.3) | 231 (7.6) | 275 (7.5) | 241 (14.7) |
| 5-19 | 42  (4.7) | 81  (5.7) | 53 (3.9) | 125 (5.6) | 131 (6.0) | 239 (6.8) | 185 (5.6) | 132 (4.4) | 155 (4.9) | 5156 (5.2) | 106 (3.5) | 150 (4.1) | 89  (5.4) |
| 20-64 | 349 (39.1) | 727 (50.7) | 671 (49.4) | 1,292 (57.6) | 1,154 (52.6) | 1,820 (52.3) | 1,613 (48.8) | 1,603 (53.3) | 1,779 (55.9) | 1,688 (56.6) | 1,866 (61.7) | 2,048 (55.8) | 671 (40.9) |
| 65+ | 157 (17.6) | 229 (16.0) | 253 (18.6) | 426 (19.0) | 409 (18.7) | 667 (19.2) | 722 (21.8) | 656 (21.8) | 793 (25.0) | 805 (27.0) | 822 (27.2) | 1,200 (32.7) | 638 (38.9) |

**Supplementary Table S2** Cross-correlation of any influenza A (any), A(H1N1) and A(H3N2) with RSV epidemic curves, 2002-2015.

| Year | Predominant subtype | RSV | Influenza A (any) | | Influenza A(H1N1) | | Influenza A(H3N2) | |
| --- | --- | --- | --- | --- | --- | --- | --- | --- |
|  |  | n | Lag (coefficient)† | n | Lag (coefficient)† | n | Lag (coefficient)† | n |
| 2002 | **H3** | 66 | **1 (0.645)** | 58 | * | 0 | 0 (0.281) | 7 |
| 2003 | **H3** | 67 | **-7 (0.674)** | 106 | -1 (0.365) | 2 | -7 (0.583) | 37 |
| 2004 | * | 56 | -15 (0.347) | 41 | * | 0 | -1 (0.586) | 1 |
| 2005 | **H3** | 110 | **-1 (0.767)** | 165 | **1 (0.710)** | 37 | **-1 (0.695)** | 59 |
| 2006 | **H3** | 93 | **-1 (0.677)** | 112 | 4 (0.589) | 3 | **1 (0.661)** | 92 |
| 2007 | **H3** | 226 | **-3 (0.730)** | 371 | **-4 (0.705)** | 80 | **-2 (0.718)** | 253 |
| 2008 | **H3** | 202 | **-10 (0.720)** | 124 | 8 (0.186) | 7 | **-8 (0.746)** | 87 |
| 2010 | **H1** | 83 | **-11 (0.629)** | 99 | **-11 (0.615)** | 78 | -14 (0.373) | 19 |
| 2011 | **H3** | 148 | **-10 (0.632)** | 172 | -5 (0.443) | 70 | **-10 (0.658)** | 97 |
| 2012 | **H3** | 162 | -3 (0.460) | 201 | 13 (0.634) | 2 | -6 (0.455) | 172 |
| 2013 | **H1/H3** | 84 | 10 (-0.428) | 160 | -12 (0.574) | 79 | 0 (-0.331) | 69 |
| 2014 | **H1/H3** | 98 | **-12 (0.673)** | 461 | **-8 (0.675)** | 199 | -13 (0.556) | 248 |
| 2015 | **H3** | 98 | -7 (0.525) | 191 | 7 (0.419) | 8 | -7 (0.543) | 171 |
| Average lag, where correlated | | | -6.0 |  | -4.4 |  | -4.0 |  |

Correlations considered moderate (>0.6) or strong (0.7) are bolded. Correlations in years with fewer than 10 cases of either virus were not considered significant.

†Lag in weeks for RSV compared to influenza A, i.e. a negative number indicates RSV preceded influenza
